# Triaging the Clinical Dilemma of a Suicidal Attempt With Sotalol Overdose Presenting With Ventricular Tachycardia, Asystole, and Torsades de Pointes

**DOI:** 10.7759/cureus.79006

**Published:** 2025-02-14

**Authors:** Manuel de la Cruz Seoane, Ambika Kapil, Pamella Morello, Sahar S Abdelmoneim, Sabas Gomez

**Affiliations:** 1 Internal Medicine, Larkin Community Hospital, South Miami, USA; 2 Osteopathic Medicine, Nova Southeastern University Dr. Kiran C. Patel College of Osteopathic Medicine, Fort Lauderdale, USA; 3 General Internal Medicine, Larkin Community Hospital, South Miami, USA; 4 Genral Internal Medicine/Cardiovascular Medicine, Assiut University Hospital, Asyut, EGY; 5 Cardiology, Larkin Community Hospital, South Miami, USA

**Keywords:** beta-blocker overdose, bradyarrhythmia, qt interval prolongation, sotalol, suicidal attempt

## Abstract

Sotalol overdose presents a significant clinical challenge due to its dual properties as a nonselective beta-blocker and potassium channel blocker, leading to life-threatening arrhythmias such as ventricular tachycardia, torsades de pointes, and asystole. The combined negative chronotropic and QT-prolonging effects of sotalol increase the risk of malignant arrhythmias, particularly in cases of overdose. We report the case of a 59-year-old female who ingested a large dose of sotalol in a suicide attempt and presented with a cascade of arrhythmias, including ventricular tachycardia, asystole, and torsades de pointes. On arrival, she was bradycardic with a heart rate (HR) of 59 beats per minute (bpm) and normotensive with blood pressure (BP) of 128/76 mmHg. However, within 24 hours, the patient deteriorated and presented with worsening bradycardia (HR: 52 bpm) and inciting hypotension (BP: 57/32 mmHg, mean arterial pressure: <60 mmHg). Despite initial resuscitative measures, including calcium gluconate, atropine, glucagon, and transcutaneous pacing, the patient progressed to asystole, requiring cardiopulmonary resuscitation and advanced cardiac life support. Given sotalol’s beta-blocking effects and potassium channel blockade, ventricular arrhythmias persisted despite the administration of amiodarone, magnesium sulfate, and dopamine for hemodynamic support. QTc prolongations of 329 ms and 463 ms were noted on telemetry, raising concern for recurrent torsades de pointes. The refractory nature of the arrhythmias necessitated emergent hemodialysis to enhance sotalol clearance despite the patient having normal renal function. Ultimately, emergent hemodialysis was initiated to enhance the clearance of sotalol. The patient’s arrhythmias resolved, and QTc prolongations were no longer noted on telemetry observation. She was subsequently discharged to a psychiatric facility without lasting cardiac or neurological deficits. This case underscores the importance of early recognition and aggressive management of beta-blocker overdose, particularly when initial therapies fail. Conventional treatments such as glucagon and magnesium sulfate were insufficient in resolving arrhythmias, likely due to sotalol’s prolonged pharmacodynamic effects and persistent QT prolongation. We highlight the successful use of hemodialysis as a definitive intervention in a patient with normal renal function, demonstrating its role in rapidly removing sotalol and preventing further toxicity. However, the availability of hemodialysis and its applicability in similar cases warrant further validation. This case provides a framework for clinicians managing severe sotalol overdose when arrhythmias persist despite standard therapies.

## Introduction

Beta-blockers are widely prescribed for managing various conditions, including hypertension, heart failure, ischemic heart disease, arrhythmias, and off-label uses for tremors, migraines, and anxiety. They are classified into cardioselective (beta-1 blockers) and non-cardioselective types (beta-1 and beta-2 blockers, some also affecting beta-3 receptors). Mechanistically, beta-blockers decrease the synthesis of cyclic adenosine monophosphate, reducing protein kinase A-mediated phosphorylation of calcium channels. This leads to decreased calcium influx, attenuating the effects of catecholamines and resulting in negative inotropic, chronotropic, and dromotropic effects.

Sotalol, a nonselective beta-blocker with class III antiarrhythmic properties, not only exerts these beta-adrenergic antagonistic effects but also inhibits the delayed rectifier potassium current (I_K_), prolonging the action potential duration and refractory period. This dual mechanism enhances nodal blockade, slows conduction through the AV node, and reduces susceptibility to reentrant arrhythmias. Unlike other beta-blockers, sotalol does not significantly affect the late sodium current (I_Na_), but its potassium channel inhibition contributes to QT prolongation, increasing the risk of torsades de pointes. Although generally well-tolerated with mild side effects, beta-blocker overdose can lead to severe cardiac instability and potentially fatal outcomes. Sotalol overdose has a unique characteristic of a higher risk of toxicity lasting more than 20 hours due to its QT-prolonging effects, which can precipitate severe arrhythmias, including ventricular tachycardia, torsades de pointes, or fibrillation.

Prior systematic review on sotalol overdose was nicely outlined in a study by Rotella et al., which examined the range of presentations and management approaches for sotalol toxicity [1]. The review highlights the necessity of timely and effective use of catecholamines, vasopressors, high-dose insulin euglycemic therapy (HIE), and veno-arterial extracorporeal membrane oxygenation (VA-ECMO) in promoting survival and hemodynamic stability. However, since these treatments were often used in combination, it is challenging to determine their individual impact. Despite the low-quality evidence and high risk of bias, the review suggests a graduated management approach: starting with fluid resuscitation, followed by the use of catecholamines or vasopressors based on the hemodynamic profile, and considering HIE as an adjunctive inotrope. In cases unresponsive to these measures, more invasive interventions such as VA-ECMO may be required. This underscores the importance of early recognition and a multidisciplinary approach to optimize outcomes in severe beta-blocker overdose [1].

We herein describe a case of a 59-year-old female who ingested 10 pills of sotalol 10 mg and 15 pills of sotalol 120 mg in a suicidal attempt. This led to a series of life-threatening arrhythmias, including ventricular tachycardia, asystole, and torsades de pointes. Her condition necessitated aggressive intervention, including urgent hemodialysis, to expedite drug clearance and restore cardiac stability. This case underscores the importance of early recognition and multidisciplinary management in severe beta-blocker overdose to prevent fatal outcomes.

## Case presentation

A 59-year-old woman presented to the ED with bodily stress syndrome (BSS) with complaints of anxiety one hour after ingesting 10 pills of sotalol 10 mg, 15 pills of sotalol 120 mg, three acetaminophen tablets, and other unidentified medications in a suicidal attempt. Per the husband, the patient exhibited paranoid ideas and disorganized thoughts for the past three days and believes they were related to recent unemployment and paranoia about being watched and ridiculed. The patient denied chest pain, palpitations, shortness of breath, vomiting, or diarrhea. Vitals on admission were as follows: blood pressure (BP) of 128/76 mmHg, pulse of 59 beats per minute (bpm), respiratory rate of 17 breaths/min, afebrile, and saturating 97% on room air. However, the rapid response was called in the ED due to acute hypotension (BP: 57/32 mmHg), mean arterial pressure: <60 mmHg, and bradycardia (pulse: 52 bpm). Physical examination was unremarkable, apart from an anxious and sad-looking patient.

Laboratory tests were largely unremarkable, except for an elevated blood urea nitrogen (BUN) of 23 mg/dL (reference: 10-20 mg/dL), a BUN/creatinine ratio of 25:1 (reference: <20:1), and a urine creatinine level of 324.8 µmol/L (reference: 123.8-229.8 µmol/L), suggesting dehydration or a stress response. Electrolyte levels were within normal limits, including serum sodium at 140 mmol/L (reference: 137-145 mmol/L), potassium at 3.6 mmol/L (reference: 3.5-5.1 mmol/L), magnesium at 2.1 mg/dL (reference: 1.6-2.3 mg/dL), total calcium at 9.5 mg/dL (reference: 8.4-10.2 mg/dL), and phosphorus at 3.6 mg/dL (reference: 2.5-4.5 mg/dL). A urine drug screen was negative, and total creatine kinase was 78 U/L (reference: 55-170 U/L). Acetaminophen levels were <10 µg/mL (reference: 10-30 µg/mL), ethanol (ETOH) was <10 mg/dL, and salicylate levels were <1.0 mg/dL (reference: 2-20 mg/dL). The measured sotalol level was 1,078 ng/mL approximately two hours after ingestion, significantly exceeding the recommended therapeutic dose of 160-320 mg per day.

**Figure 1:**
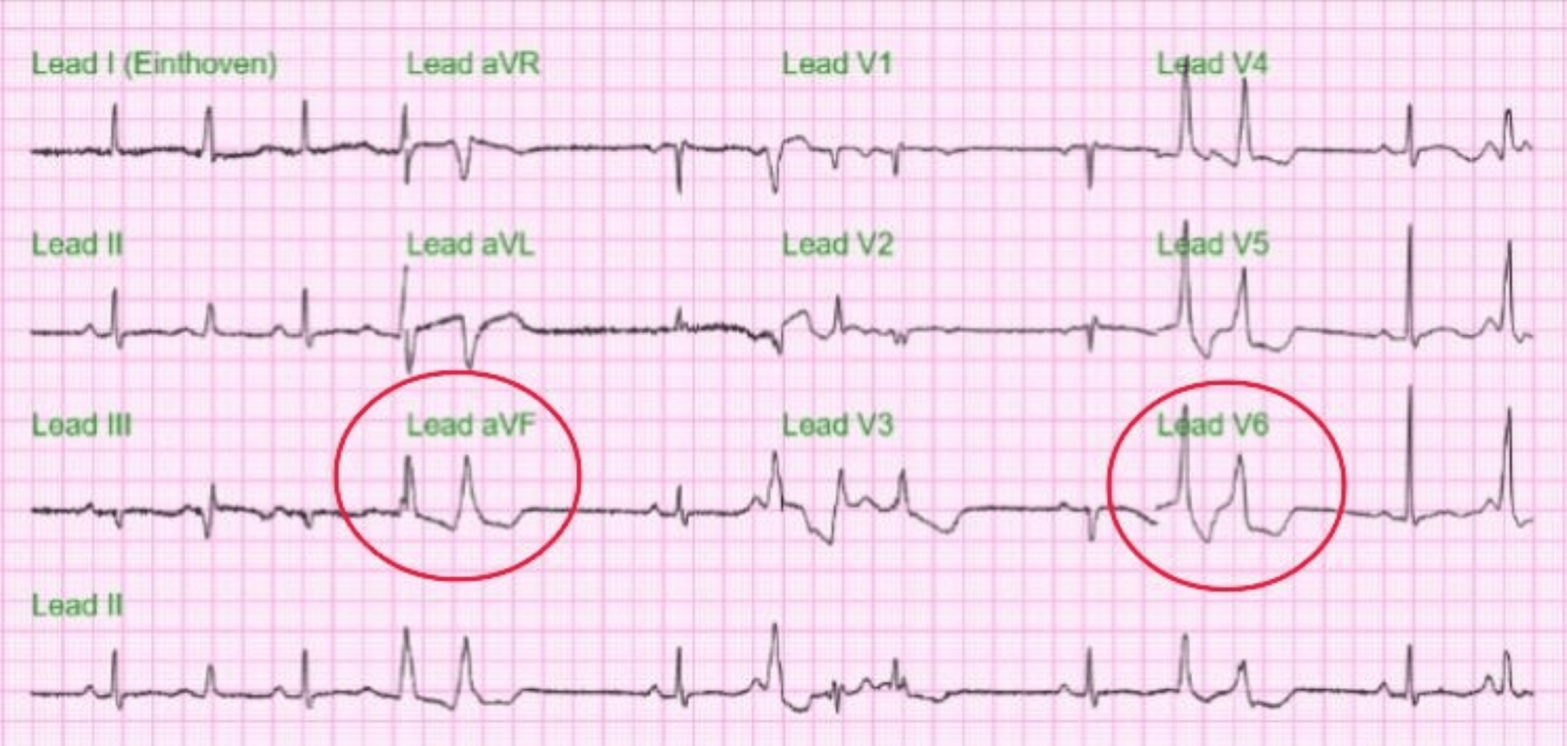
ECG during admission Hospital day 1: Sinus rhythm with frequent ventricular premature beats at times occurring as couplets diffuse nonspecific ST-T wave changes (red circles).

**Figure 2:**
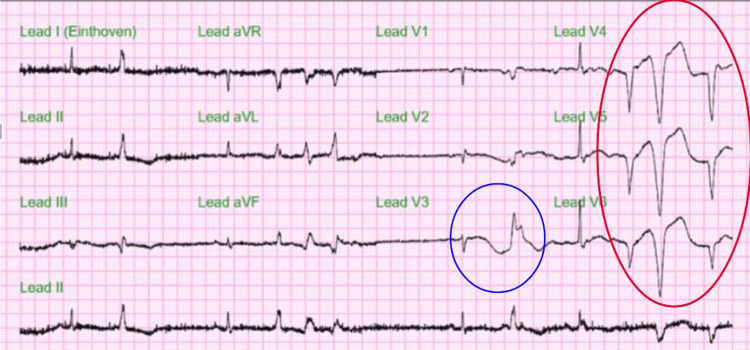
Follow-up ECG on hospital day 1 Sinus arrhythmia and frequent ventricular premature beats (blue circle). In addition, there are diffuse nonspecific ST-T wave changes (red circle).

The initial ECG in Figure 1 showed frequent premature ventricular contractions (PVC), sometimes occurring as diffuse couplets with nonspecific ST-T wave changes, and the corrected QT interval (cQT) was 329 ms. Follow-up ECG later on the same day showed sinus arrhythmia and frequent ventricular premature beats along with diffuse nonspecific ST-T wave changes of the ST segment and prolongation of cQT interval of 463 ms (Figure 2).

**Figure 3:**
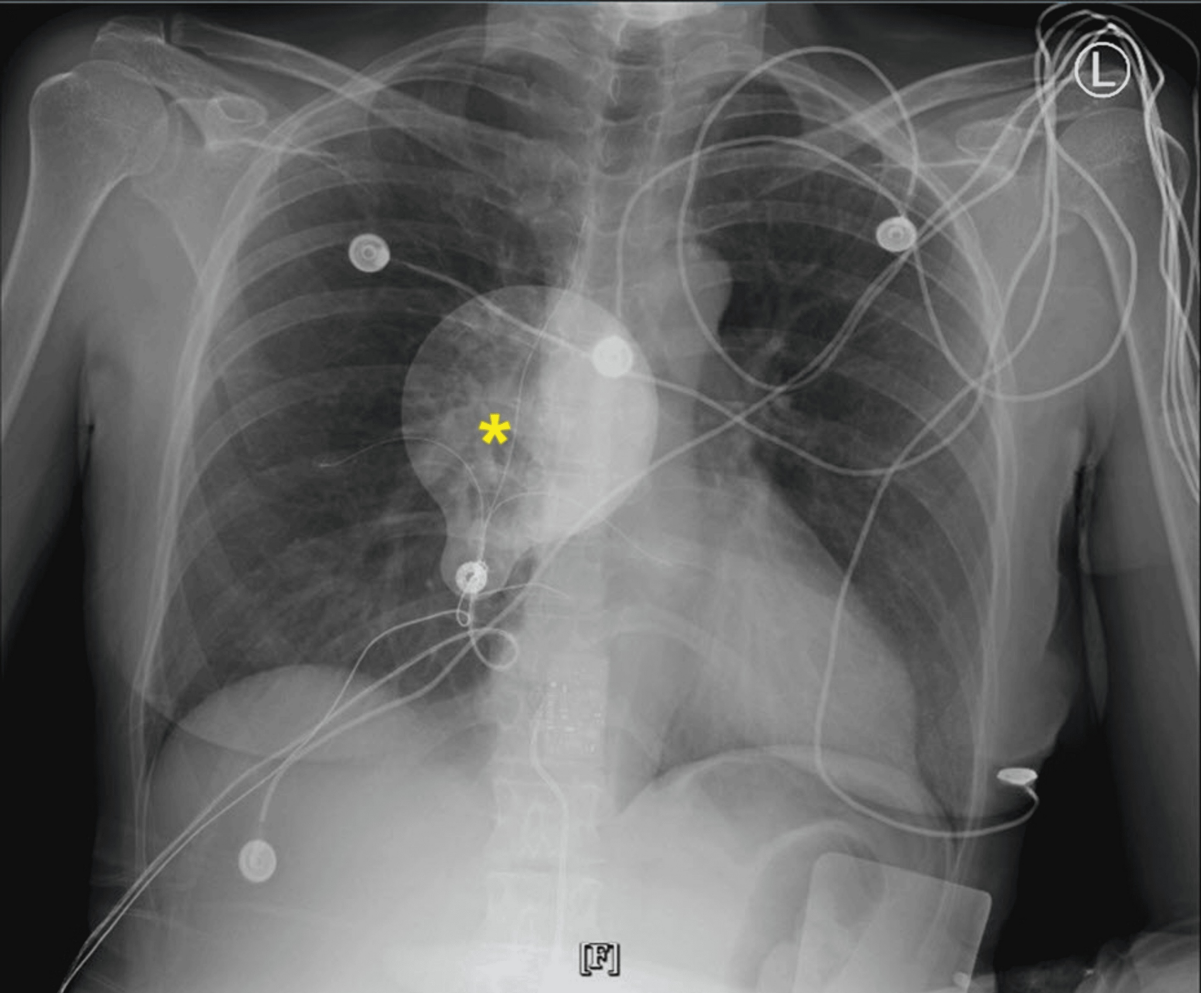
Chest X-ray on admission No focal pulmonary consolidations or significant pleural effusions. The asterisk (*) denotes the external defibrillator pad.

**Figure 4:**
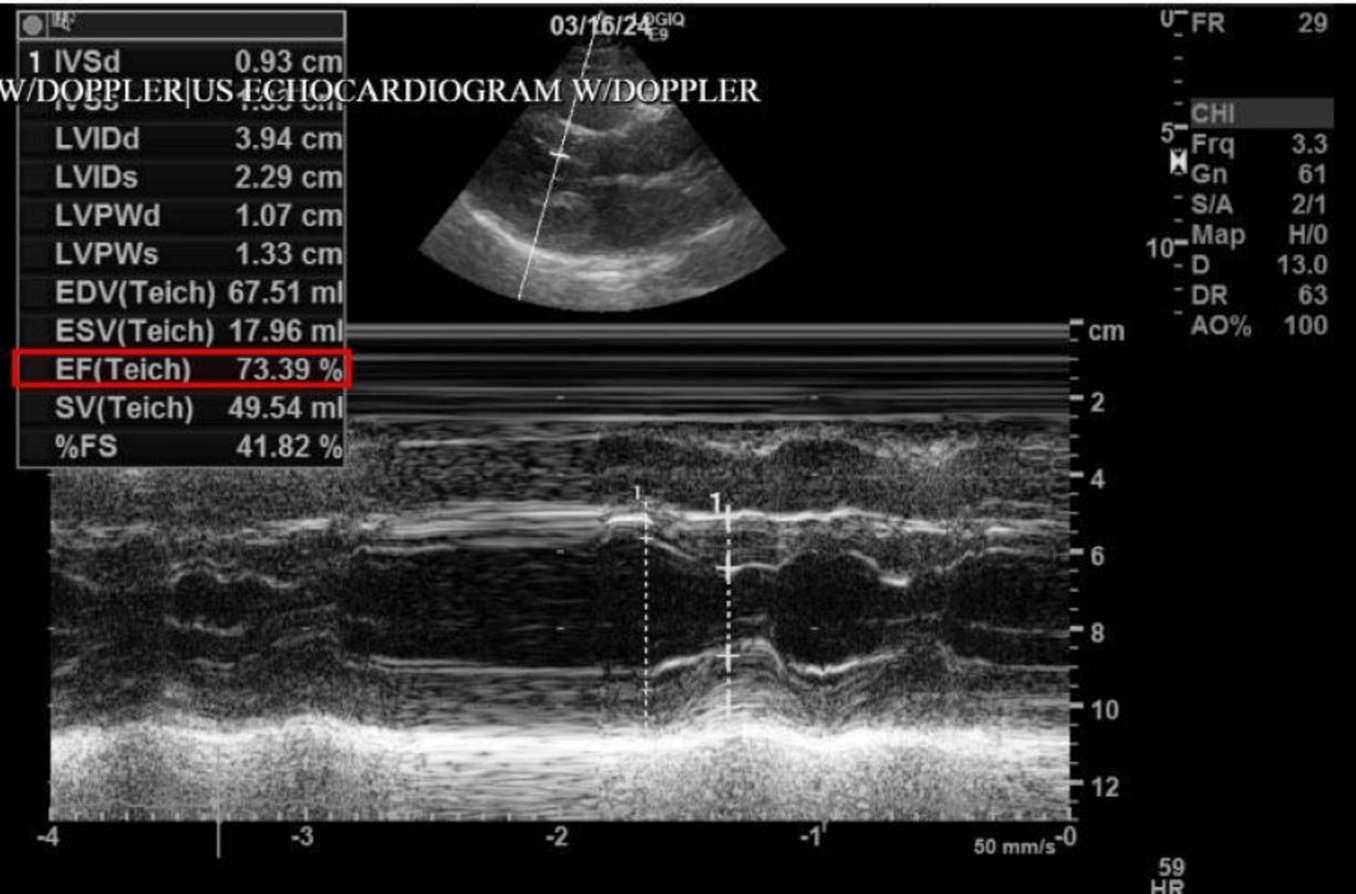
Transthoracic echocardiogram M-mode of the left ventricle showing preserved contractility of the LV. Preserved systolic function, indicated by an LVEF within the 54-74% range (red rectangle). LVEF, left ventricular ejection fraction

Chest X-ray, as shown in Figure 3, was unremarkable. A transthoracic echocardiogram (Figure 4) was performed and showed a left ventricular ejection fraction of 54-74%, trace tricuspid and mitral regurgitation, mitral valve leaflets thickened, and a dilated inferior vena cava with <50% collapse with inspiration, no other abnormalities seen. CT was also performed and revealed no evidence of acute intracranial hemorrhage, midline shift, or mass effect. In the ED, the patient was given calcium gluconate 1 g IV push (IVP), atropine sulfate 0.4 mg IVP Q2M (total of three doses), and glucagon 10 mg IV. Furthermore, a transcutaneous pacemaker was placed on the patient during admission as a standby tool in the case of bradyarrhythmic events. The patient was admitted to the intensive care unit on telemetry for beta-blocker intoxication, hypotension, bradycardia, and suicidal attempts. A multidisciplinary team of intensivists and cardiologists was involved in patient care as well as poison control. The patient required dopamine 800 mg/ 500 ml (800 mg IV, titrated “pro re nata” (PRN)) and lactated ringers 1,000 mL IV continuous 125 mL/hr for hemodynamic support. On the ICU monitor, the patient experienced arrhythmias ranging from bradycardia to PVC’s to short runs of tachycardia; however, hemodynamically, it was becoming stable. A cardiac defibrillator was on standby PRN for hemodynamically unstable ventricular tachycardia. Later in the day, the patient experienced episodes of frequent runs of ventricular tachycardia, for which the patient received amiodarone bolus (300 mg total), magnesium 2 mg IV, and another dose of glucagon 10 mg IV was given. Prophylactic anticoagulation with enoxaparin was initiated. The patient was Baker Acted by the psychiatry team and placed with a 24-hour sitter and continuous monitoring for vitals. Psychotropic treatment was held until cardiac stabilization due to its effects on QTc prolongation.

**Figure 5:**
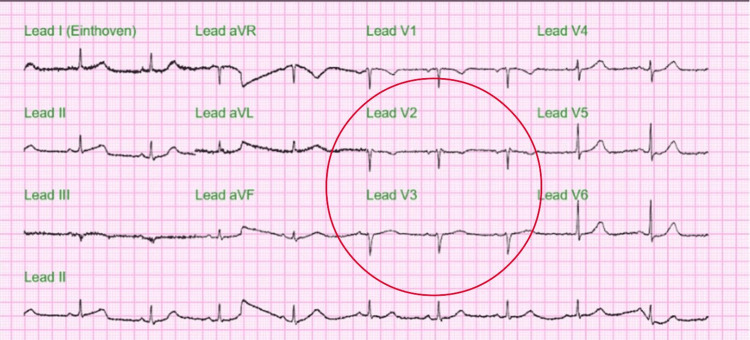
Hospital day 2 follow-up ECG Follow-up ECG showing bradycardia of 57 beats/min to the progression of the R wave in V2 and V3, with no significant ST-T change (red circle).

On ICU day 2, a Code Blue was activated due to progression from arrhythmia to asystole requiring cardiopulmonary resuscitation, to torsades de pointes and hemodynamically unstable ventricular tachycardia (Figure 5). The patient was electrically cardioverted, and amiodarone 300 mg IVP and magnesium 2 g were given. At this point, the nephrology team recommended emergent hemodialysis to help clear the drug. The general surgery team performed Urgent Trialysis catheter placement, and hemodialysis was received on days 2 and 3, along with potassium replacement. The interventions stabilized the patient and continued treatment with PRN Atropine and potassium chloride. Amiodarone was discontinued to prevent bradycardia. On day 5, she had no recurrence of arrhythmias. Per psychiatry recommendations, venlafaxine 75 mg per os daily and trazodone 50 mg at night were initiated. The patient was transferred out of the ICU on day 6 and discharged to a psychiatric facility on day 7 with no cardiac or neurological aftereffects. Additionally, the patient was educated that sotalol is now an absolute contraindication indefinitely. Furthermore, she was instructed on how to follow up with a psychiatrist and cardiologist after in-hospital treatment.

## Discussion

Sotalol is a nonselective beta-blocker that lacks intrinsic sympathomimetic activity and is distinct for its capacity to notably increase the duration of the cardiac action potential due to its competitive inhibition of the potassium current [2,3]. It is primarily used to manage conditions such as supraventricular arrhythmias, severe ventricular arrhythmias, and atrial fibrillation or flutter [4].

Due to sotalol’s pharmacodynamic properties, including its delayed clearance and prolonged receptor activity, overdose cases present a significant challenge as patients may initially stabilize only to experience a “second wave” of toxicity hours later. This delayed toxicity often manifests as life-threatening arrhythmias, such as ventricular tachycardia, due to the drug’s continued action as a potassium channel blocker and a non-cardioselective B-blocker. The concentration of sotalol directly correlates with the degree of QTc prolongation, exacerbating the risk of arrhythmias. Higher doses and plasma concentrations lead to more pronounced QTc prolongation, increasing the likelihood of torsades de pointes [5]. Early management typically involves magnesium sulfate to counteract QT prolongation, isoproterenol to stabilize heart rate, and pacing to address bradyarrhythmias. Cases of severe ventricular tachyarrhythmias, particularly within the first 20 hours of ingestion, were seen in five of six patients in these studies, underlining the heightened risk during this critical window. These findings further support the necessity of aggressive and early interventions to mitigate life-threatening arrhythmias [6,7].

The most concerning sign of toxicity is its dose- and concentration-dependent effect on QTc prolongation. At doses exceeding 320 mg, the risk of torsades de pointes increases to 5%. Given this, before administering sotalol, it is generally advised to obtain a baseline ECG, check serum potassium and magnesium levels, and calculate creatinine clearance. This drug may predispose the patient to cardiac arrhythmias by promoting a mechanism that contributes to the elevation of the ST segment [1,5,8]. Furthermore, as seen in this case and others in the literature, QTc prolongation often becomes refractory to initial stabilization efforts, such as pacing and magnesium administration. High-dose insulin therapy, supplemented with glucose, has shown promise in cases of severe beta-blocker poisoning, particularly when conventional treatments such as atropine, glucagon, or calcium prove insufficient [9]. This approach improves cardiac output without increasing myocardial oxygen demand, complementing other therapies like dialysis to accelerate drug clearance. The risk of arrhythmias like torsades de pointes may necessitate advanced interventions to break the cycle of recurrent instability [10].

Notably, the patient was asymptomatic upon arrival to the emergency room yet underwent a hypotensive crisis within three hours of the initiation of the drug overdose. In the case of a patient with normal kidney function for their age, as in this case, the role of emergent dialysis in sotalol overdose may seem less intuitive but remains significant in reducing mortality [11]. Sotalol is primarily excreted by the kidneys, and even with normal renal function, the half-life of the drug can extend to seven to 15 hours, prolonging toxicity [12]. The body’s ability to clear the drug may not be fast enough to prevent recurrent arrhythmias or severe QTc prolongation. Hence, dialysis accelerated the clearance of the drug, effectively reducing plasma sotalol levels more rapidly than natural renal elimination [12]. This was particularly critical in this patient, who later developed severe QTc prolongation despite early treatment with other interventions [13]. By expediting drug removal, dialysis helped halt toxicity progression and provided a potential solution to reversing life-threatening cardiac effects [14].

While dialysis is traditionally reserved for patients with renal impairment or extremely dialyzable drugs, this case would explore dialysis as a proactive measure in patients with normal renal function to prevent delayed-phase toxicity. Real-time monitoring of plasma sotalol levels and cardiac stability informed the decision for early dialysis, providing new insights into an often-underutilized intervention. This unique perspective could offer guidance to clinicians on when to consider dialysis, even in patients whose renal function remains intact, thereby extending the range of therapeutic options for managing severe sotalol overdoses.

## Conclusions

This case highlights the importance of emergent dialysis as a proactive intervention for managing severe sotalol overdose, even in patients with normal renal function. While traditionally reserved for renal impairment, dialysis played a critical role in rapidly clearing sotalol and halting the progression of toxicity in this case. The real-time decision to initiate dialysis, guided by monitoring of plasma sotalol levels and cardiac stability, offers a unique perspective on overdose management. Magnesium was utilized as a key therapeutic measure to stabilize the myocardium and mitigate arrhythmic risk. However, agents like isoproterenol were avoided due to concerns about its proarrhythmic potential and its tendency to exacerbate QT prolongation. This case highlights the importance of expanding therapeutic options for clinicians treating high-risk sotalol overdoses and underscores the need for further research or case studies to validate the proactive use of dialysis in similar scenarios.
